# The Fc Receptor Polymorphisms and Expression of Neutrophil Activation Markers in Patients with Sickle Cell Disease from Western India

**DOI:** 10.1155/2013/457656

**Published:** 2013-09-29

**Authors:** Harshada K. Kangne, Farah F. Jijina, Yazdi M. Italia, Dipti L. Jain, Anita H. Nadkarni, Maya Gupta, Vandana Pradhan, Rati D. Mukesh, Kanjaksha K. Ghosh, Roshan B. Colah

**Affiliations:** ^1^Department of Hematogenetics, National Institute of Immunohaematology (Indian Council of Medical Research), 13th floor, New Multistoried Building, KEM Hospital Campus, Parel, Mumbai 400012, India; ^2^Haematology Department, KEM Hospital, Parel, Mumbai 400012, India; ^3^Valsad Raktadan Kendra, Valsad 396001, India; ^4^Department of Pediatrics, Government Medical College, Nagpur 440003, India

## Abstract

*Objective*. Sickle cell disease has variable clinical manifestations. Activation of neutrophils plays an important role in the initiation and propagation of vaso occlusive crises which can be analysed by determining the expression of neutrophil antigens such as CD16, CD32, and CD62L. The common Fc**γ**R polymorphisms (Fc**γ**RIIA and Fc**γ**RIIIB) are considered to influence clinical presentation. This study focuses on distribution of Fc**γ**R polymorphisms and their association with neutrophil activity among the patients from western India. *Methods*. In this paper 127 sickle cell anemia patients and 58 patients with sickle-**β**-thalassemia (median age 12 ± 8.58 years) with variable clinical phenotypes along with 175 normals were investigated. Fc**γ**Rs polymorphisms were analysed by RFLP and AS-PCR. Activation of neutrophils was measured by flow cytometry. *Results*. The genotypic frequency of the H/R genotype of Fc**γ**RIIA and the NA1/NA1 genotype of Fc**γ**RIIIB was significantly decreased in patients compared to normals (*P*-0.0074, *P*-0.0471, resp.). We found a significant difference in the expression of CD32 and CD62L among the patients as against normals. A significantly higher expression of CD32 was seen in the milder patients with the H/H genotype (*P*-0.0231), whereas the expression of CD16 was higher in severe patients with the NA2/NA2 genotype (*P*-0.0312). *Conclusion*. The two Fc**γ**R polymorphisms had significant association with variable phenotypes of sickle cell disease. The expression of CD62L decreased in our patients indicating activation of neutrophils.

## 1. Introduction

Sickle cell disease is an inherited hemoglobin disorder with an extremely variable clinical presentation ranging from a very severe disease with multisystem involvement to a relatively asymptomatic condition. The clinical manifestations include painful vaso occlusive crises, chronic hemolysis, frequent infections and various acute and chronic complications which can lead to organ damage, disability or premature death. The main cause of sickle cell associated morbidity and mortality is vascular occlusion. Several genetic factors also contribute to this phenotypic variability. The genetic modifiers within the globin gene clusters have been extensively studied in different populations; however, the role of various nonglobin gene modifiers is now being investigated [1–7].

The Fc receptors on the cell surface play an important role in various pathophysiological events. The common Fc*γ*R polymorphisms are known to alter ligand specificity and receptor mediated effector functions of leucocytes. The Fc*γ*RIIA gene is expressed in two polymorphic forms, R131 and H131, which differ by replacement of histidine by arginine at position 131; whereas Fc*γ*RIIIB exists in two allelic forms, as NA1 and NA2 which differ in five nucleotides (nucleotides 141, 147, 227, 277, and 349) which leads to changes in four amino acids at positions 36, 65, 82, and 106; one of which is a silent mutation. An additional polymorphism of the Fc*γ*RIIIB is the HNA-1c (SH) antigen which is associated with the Fc*γ*RIIIB-SH allele. NA2 and SH allele differ by a single nucleotide mutation at position 60 [8, 9].

This study mainly focuses on role of Fc gamma receptor (Fc*γ*R) polymorphisms among sickle cell disease patients from western India.

## 2. Materials and Methods

### 2.1. Study Population

The study population consisted of 185 sickle cell disease (SCD) patients (103 males and 82 females) of whom 127 had sickle cell anaemia (HbS homozygous) and 58 had HbS-*β*-thalassemia with a median age of 12 ± 8.58 years. 175 age and sex matched normals were also included. All the patients were on folic acid supplements. None of them were on hydroxyurea therapy. The study was approved by the Institutional Ethics Committee. (Ref. number IIH/IEC/13-2009).

### 2.2. Clinical Evaluation

All the patients were evaluated clinically. A standard questionnaire was filled for recording the clinical presentation, previous history, and other demographic information. Depending upon frequency of painful crises per year, blood transfusion requirements, acute chest syndrome, avascular necrosis, stroke, spleen size, and need for hospitalization, the patients were given scores according to the scoring system used earlier [10] with some modifications. This scoring system was validated against classification of the patients as severe or mild by a haematologist and/or a pediatrician with considerable experience in managing patients with sickle cell disease. Patients with a total score of less than or equal to 10 were considered as mild and those with a score of more than 10 were considered as severe (Table 1).

### 2.3. Blood Samples

Venous blood samples (4-5 mL) were collected from healthy volunteers and patients into ethylenediaminetetraacetic acid (EDTA) anticoagulant vials after obtaining an informed consent. The blood samples were immediately processed for flow cytometric analysis.

### 2.4. Hematologic Analysis

Complete blood count was measured by automated cell analyser (SYSMEX K-1000, Kobe Japan). HbA2, HbF, and HbS levels were estimated by HPLC on the Variant Haemoglobin Testing System (Biorad Laboratories, Inc, Hercules, CA, USA).

### 2.5. Monoclonal Antibodies

The following monoclonal antibodies (m-Abs) were used: anti-CD16 FITC, anti-CD32 PE, and anti-CD62LPE-CyTM^5^ (Becton Dickinson, San Joes, CA, USA).

### 2.6. Immunophenotyping Analysis

The blood samples were processed using the stain-lyse-wash technique. 50 *μ*L of blood was incubated with 5 *μ*L anti-CD16 FITC and 15 *μ*L of anti-CD32 PE and 5 *μ*L of anti-CD62L PE-CyTM^5^ for 20 minutes at room temperature in the dark. Erythrocytes were lysed by using 1 ml of 1% FACS-LYSE solution for 10 minutes. Repeated washes were given with phosphate-buffered saline (PBS). Finally the cells were suspended in 300 *μ*L of PBS. The unstained tube was prepared similarly but without adding mAbs. Flow cytometric analysis was performed with a FACScan flow cytometer (Becton Dickinson). Ten thousand events in the neutrophil region were collected and analyzed using the CELL-QUEST software (Becton Dickinson).The expression of CD16, CD32, and CD62L was obtained in terms of median fluorescence intensity (MFI) after taking the ratio of the stained to unstained population.

### 2.7. Molecular Analysis

Extraction of DNA was done by the standard phenol-chloroform method. Analysis of XmnI polymorphism and haplotype analysis of the beta globin cluster was done by restriction enzyme analysis [11]. The Fc*γ*RIIA polymorphism was studied by restriction enzyme digestion [12], whereas the Fc*γ*RIIIB genotyping was done by allele specific PCR as described earlier [8].

### 2.8. Statistical Analysis

The descriptive data were given as mean ± standard deviation (SD). The differences between the patients and normals were evaluated using the Student's *t* test. The differences between the calculated gene frequencies were determined using the chi-square test and odds ratio. Differences between the groups were considered to be significant when *P* ≤ 0.05.

## 3. Results

All the sickle cell disease patients included in the study showed variable clinical phenotypes. Based on the scoring system, 110 patients were classified as mild and 75 were severe. Among the mild patients, 11 of the 49 patients who had a score of 7 were asymptomatic. Most of our patients had history of painful crises with variable severity from mild to severe, often requiring hospitalization followed by history of jaundice and fever. Forty six patients had severe crises (>3 episodes/year), among them 29 were HbS-homozygous patients and 17 were HbS-*β*-thalassemia patients. 20 patients required multiple blood transfusions (>3 times/year). Eight patients had history of acute chest syndrome (5 HbS-homozygous and 3 HbS-*β*-thalassemia) whereas 9 patients presented with avascular necrosis (6 HbS-homozygous and 3 HbS-*β*-thalassemia). Only1 patient had a history of stroke. 36 patients had splenomegaly. (Spleen: Mean ± SD = 9.38 ± 4.9 cm.) Thirty patients had history of infection mainly malaria, typhoid, pneumonia, and hepatitis. Two patients had history of AFB infection.

Hematological investigations showed that the haemoglobin levels and red cell indices were significantly decreased in sickle cell disease patients as compared to normals (*P* < 0.0001). The HbA2 levels were significantly increased in HbS-*β*-thalassemia patients as compared to HbS-homozygous patients (*P* < 0.0001) (Table 2).

85.32% of the sickle homozygous patients and 43.1% of the sickle-*β* thalassemia patients showed the presence of the XmnI polymorphism (+/+). Among the sickle homozygotes and sickle-*β* thalassemia patients, the milder patients showing presence of XmnI (+/+) or XmnI (+/−) polymorphism had higher HbF levels (Mean ± SD: 16.04 ± 3.12) than the severe group of patients (Mean ± SD: 13.33 ± 4.94). The mean HbF levels were higher in patients showing homozygosity for the presence of the XmnI polymorphism (+/+).

The *β*-globin cluster haplotype was studied in 15 sickle homozygous patients showing the XmnI (+/−) polymorphism. 15 chromosomes were linked to the Arab-Indian haplotype and 6 atypical haplotypes were observed with varying frequencies. Out of these 6 atypical haplotypes, 4 have been reported earlier, whereas 2 haplotypes (+ −− − − − + + − and + − − − − − − − +) are novel ones. Some of the atypical haplotypes were associated with lower HbF levels (HbF: 6.2% to 9.8%). 

The expression of CD32 was significantly increased and the level of CD62L was significantly decreased in both the mild and severe patients as compared to normals. (*P* < 0.0001). However, among the patient group (milder and severe) no significant differences were observed (Table 3).

The Fc*γ*RIIA genotypic frequency of the H/R genotype was significantly decreased in the patients as compared to normals (*P*-0.0074). The genotypic frequency of the R/R genotype in the milder group of patients (*P*-0.0471) and that of H/H genotype in the severe group of patients (*P*-0.0172) was significantly increased as compared to normals. The Fc*γ*RIIIB NA1/NA1 genotype was significantly decreased in patients as compared to normal (*P*-0.0006) (Table 4).

When we tried to correlate genotypic frequencies of Fc*γ*RIIA and of Fc*γ*RIIIB with the expression of CD32 and CD16, we found significantly higher expression of CD32 in the milder patients with the H/H genotype as compared to severe patients (*P*-0.0231). Similarly severe patients with the NA2/NA2 genotype of Fc*γ*RIIIB showed higher expression of CD16 as compared to milder patients (*P*-0.0312) (Table 5).

Ninety seven of our patients had history of vaso occlusive crises (VOC) against 51 patients showing no episode of VOC. When we compared the expression of CD16, CD32 and CD62L among patients with vaso occlusive crises there were no significant differences observed in the expression of CD16 and CD32 among these groups; however, CD62L level was significantly different (*P*-0.019) (Table 6).

## 4. Discussion

Sickle cell disease is widely prevalent in many tribal populations as well as in a few nontribal populations in India with a very variable clinical presentation [13]. During the last several years, many studies have investigated the role of different genetic modifiers that maybe involved in the variable phenotypic presentation of sickle cell disease, and variations in a few genes have been associated with the clinical course of the disease. Increased fetal hemoglobin levels associated with a mutation in the gamma gene where the sickle mutation is linked to the Arab Indian haplotype are known to influence the clinical severity. We studied the Fc receptor polymorphisms and activation of neutrophils and their association with each other in sickle cell disease patients from western India.

The G*γ*-158 (C→T) polymorphism is a modulator of the severity of sickle cell anemia. The XmnI restriction site at −158 position of the G*γ*-gene is associated with increased expression of the G*γ*-globin gene and higher production of HbF. Rahimi et al. had studied the level of HbF and *γ*G-gene expression and their association with haplotypes and XmnI polymorphism. The Hb F level was significantly higher in SS patients with one or both sickle genes linked to the Arab-Indian haplotype as compared to the Bantu, Benin, and Cameroon haplotypes [14]. Earlier studies on Indian patients have also shown that the HbF levels in sickle homozygotes were much higher with the Arab Indian haplotype than with other haplotypes. According to the study by Bhagat et al. the presence of the XmnI (+/+) site in sickle homozygotes and sickle cell trait individuals was associated with increased HbF synthesis (*P* < 0.0001) while the presence of XmnI site (+/−) in SS patients compared to individuals with XmnI (−/−) had no difference on HbF levels [15]. Similar results were observed in our study. Both HbS-homozygous and HbS-*β*-thalassemia patients had significantly higher HbF levels with presence of the G*γ* gene mutation than those without this mutation (*P* < 0.0001). As increased HbF levels are associated with a milder clinical course of sickle cell disease, patients with presence of the XmnI polymorphism from our milder group had higher HbF levels (Mean ± SD: 16.04 ± 3.12) than the severe group of patients (Mean ± SD: 13.33 ± 4.94).

The HbS gene is found on a genetic background of five major *β*-globin gene cluster haplotypes [14]. Previous studies have shown that carriers of the HbS gene on the Senegal or Arab-India haplotype usually have the highest HbF levels and PCV and the mildest clinical course [16]. According to the study done by Mukherjee et al., on sickle cell anaemia patients from Gujarat and Maharashtra in western India, 91.5% of the *β* ^*s*^ chromosomes were linked to the Arab-Indian haplotype and showed HbF levels of 15.5 ± 4.5. The remaining 8.5% of the chromosomes were associated with 6 different atypical haplotypes [16]. 18% of our sickle homozygous patients who were XmnI (+/−) showed that one of their sickle chromosomes was not linked to the Arab-Indian haplotype. Six atypical haplotypes were found in our study of which 4 were reported earlier by Mukherjee et al., [17] whereas 2 were novel ones.

Several studies have been carried out to look for the genotypic frequencies of Fc*γ*RIIA and Fc*γ*RIIIB in different diseases such as colorectal cancer, chronic periodontal disease, and systemic lupus erythematous [12, 18, 19]. Kuwano et al. determined allelic polymorphisms of Fc*γ*RIIA and Fc*γ*RIIIB among 263 unrelated Brazilian subjects, including Amazon Indians, blood donors, and sickle cell disease patients. They found that the distribution of the Fc*γ*RIIIB allele was significantly different in Amazon Indians compared to Brazilian blood donors or African Brazilian patients with sickle cell disease [8]. Flesch et al. also reported similar results to that of the phenotypic data of blood donors [9].

The 131R allele of Fc*γ*RIIA was predominantly reported in our Indian population. It has been conjectured that as the 131H allele represents the human leucocyte Fc receptor allotype capable of binding to IgG2, it might have been subjected to strong evolutionary forces, in order to confer protection against various inflammatory disorders. This may be the reason behind the variable genotype frequencies of the R131H polymorphism among different ethnic groups, especially Caucasians and Asians [20]. The frequency of the R/R genotype was significantly increased in our milder group of patients as compared to normals whereas the H/H genotype frequency was significantly increased in the severe group suggesting that with evolutionary forces the R allele may have evolved to play a protective role against inflammatory disorders. The frequency of the NA1/NA1 genotype of Fc*γ*RIIIB was significantly decreased in our patients. The NA2 allele was found to be predominant in our group of patients. Similar results were reported by Pradhan et al. in Indian SLE patients suggesting it's influence on inflammatory disorders [19].

Lard et al. had studied the activated state of neutrophils by analysing levels of expression of various neutrophil antigens such as CD62L, CD11b, CD66b, and Fc receptors [7]. The levels of CD32 and CD62L were significantly different in patients as compared to normals (*P* < 0.0001). The level of CD32, was significantly increased and that of CD62L was significantly decreased in patients as compared to normals. Similar findings were observed for the expression of CD32 and CD62L among our mild and severe patient groups against normals (*P* < 0.0001). However, no significant differences in expression of CD16, CD32 and CD62L were observed between mild and severe patients. The expression of CD62L on neutrophils was decreased in our patients indicating that CD62L (L-Selectin), a membrane-bound molecule, is more likely to be a very sensitive marker, which gets shed off during activation of neutrophils.

We did not find any change in the CD16 expression on the neutrophils in sickle cell disease patients. This unaltered expression of CD16 during crises may be because of activation induced shedding of CD16 and at the same time upregulation of CD16 expression by fusion of secretory vesicles with the plasma membrane as has been suggested earlier [9]. In contrast to our study, Okpala et al. found increased expression of L-selectin by lymphocytes and neutrophils in sickle cell disease patients. They have suggested that high steady-state expression of L-selectin by leucocytes predisposes to severe manifestations [21].

Comparison of genotypic frequencies of Fc*γ*RIIA and of Fc*γ*RIIIB with the expression of CD32 and CD16, respectively, showed higher expression of CD32 in the milder patients with the H/H genotype, whereas severe patients with the NA2/NA2 genotype of Fc*γ*RIIIB had higher expression of CD16 as compared to milder patients, indicating that a significant association exists between Fc receptors and these CD markers. We also found that the expression of CD62L was significantly different in sickle cell disease patients with vaso occlusive crises as compared to those without crises indicates that neutrophils are activated in sickle cell disease patients, especially, during vaso occlusive crises. As shown earlier by Faldon et al., [22], these activated neutrophils may lead to increased adherence to the endothelium in the microcirculation of sickle cell disease patients which further increases the risk for vaso occlusive crises due to a subsequent delayed passage of RBCs and WBCs in the microcirculation.

To conclude, our data suggests that two Fc*γ*R polymorphisms have significant association with variable phenotypes of sickle cell disease. The difference in the genotype frequency of H131R of Fc*γ*RIIA is more likely because of our different ethnic origin. The R131 allele of Fc*γ*RIIA might have evolved to play a protective role against inflammatory disorders. The NA2 allele of Fc*γ*RIIIB plays an important role in susceptibility of disease manifestations. The expression of CD62L (L-Selectin), a membrane-bound molecule on neutrophils was decreased in our patients indicating activation of neutrophils which may lead to increased adherence to the endothelium in the microcirculation. This reconfirms that neutrophils may play an important role in the initiation and propagation of vaso occlusive crises.

## Figures and Tables

**Table 1:** Scoring system for clinical evaluation of patients.

Clinical parameters	Score				
	1	2	3	4	5
Vaso occlusive crisis (VOC)/yr	0 to 1	>2 to 3	>4 to 5	>6 to 8	>9 to 12
Hospitalizations due to severe VOC/yr	0	1 to 2	3 to 4	>5	
				
Blood transfusions (BT)/yr	0	1 to 2	3 to 5	>5	
Acute chest syndrome (ACS)	No			Yes	
Stroke	No			Yes	
Avascular necrosis of femur (ANF)	No	Yes			
Spleen	Just palpable	3–4 cm	5–7 cm	Big spleen	

**Table 2:** Hematological parameters among sickle cell disease patients and normal.

	HbS-homozygous (127)	HbS-*β*-thalassemia (58)	Total (185)	Normals (75)	*P Value (Total patients versus normals)
WBC ∗ 10^3^/mL	12.11 ± 7.93	11.20 ± 6.25	11.82 ± 7.43	6.64 ± 1.46	<**0.001**
RBC ∗ 10^6^/mL	3.25 ± 0.96	3.89 ± 0.98	3.46 ± 1.01	5.00 ± 0.49	<**0.0001**
HB (g/dL)	8.46 ± 2.20	8.5 ± 2.01	8.47 ± 2.14	14.62 ± 1.63	<**0.0001**
HCT (%)	25.68 ± 6.45	26.7 ± 5.95	25.99 ± 6.29	42.86 ± 3.91	<**0.0001**
MCV (fL)	79.93 ± 12.09	69.27 ± 7.67	76.48 ± 11.92	85.86 ± 7.42	<**0.0001**
MCH (pg)	26.58 ± 4.22	22.06 ± 2.52	25.12 ± 4.31	29.31 ± 3.28	<**0.0001**
MCHC (g/dL)	32.91 ± 2.01	32.91 ± 8.63	32.91 ± 5.34	33.48 ± 3.41	0.39
RDW (%)	20.41 ± 4.58	22.29 ± 4.45	21.02 ± 4.61	14.11 ± 1.23	<**0.0001**
PLT ∗ 10^3^/mL	321.68 ± 174.49	296.89 ± 142.09	313.67 ± 164.8	269.42 ± 53.96	0.02
HbA2 (%)	3.05 ± 0.79	5.09 ± 0.96	3.71 ± 1.27	2.79 ± 0.28	<**0.0001**
HbF (%)	18.97 ± 9.5	19.14 ± 8.33	19.02 ± 9.12	0.30 ± 0.36	<**0.0001**
HbS (%)	72.06 ± 10.12	68.21 ± 9.29	70.81 ± 9.99		

*Student's *t* test analysis comparing hematological parameters among patients and normal.

**Table 3:** Expression of neutrophil markers among sickle cell disease patients.

	SCD-mild (90) (Mean ± SD)	SCD-severe (61) (Mean ± SD)	SCD-total (151) (Mean ± SD)	Normals (72) (Mean ± SD)	*P value (SCD-Total versus normals)	*P value (SCD-Mild versus normals)	*P value (SCD-Severe versus normals)	*P value (SCD-Mild versus SCD Severe)
CD16 (MFI)	178.07 ± 90.17	169.84 ± 92.61	174.75 ± 90.95	170.06 ± 94.89	0.72	0.58	0.98	0.58
CD32 (MFI)	30.84 ± 21.73	27.03 ± 21.03	29.30 ± 21.46	13.11 ± 7.05	<**0.0001**	<**0.0001**	<**0.0001**	0.28
CD62L (MFI)	85.08 ± 45.79	100.94 ± 62.24	91.49 ± 53.43	161.94 ± 73.49	<**0.0001**	<**0.0001**	<**0.0001**	**0.07**

*Student's *t* test analysis comparing expression levels in different groups.

**Table 4:** Distribution of the H and R alleles of Fc*γ*RIIA and NA1 and NA2 alleles of Fc*γ*RIIIB in sickle cell disease patients.

Group	Number of cases	Fc*γ*RIIA			Fc*γ*RIIIB		
		H/H Number (%)	H/R Number (%)	R/R Number (%)	NA1/NA1 Number (%)	NA1/NA2 Number (%)	NA2/NA2 Number (%)
SCD-mild	110	20 (18.2)	36 (32.7)	54 (49.1)	2 (1.8)	55 (50)	53 (48.2)
SCD-severe	75	23 (30.7)	25 (33.3)	27 (36)	0 (0)	48 (64)	27 (36)
SCD-total	185	43 (23.3)	61 (32.9)	81 (43.8)	2 (1.1)	103 (55.7)	80 (43.2)
Normals	175	28 (16)	82 (46.9)	65 (37.1)	22 (12.6)	90 (51.4)	63 (36)
*P value SCD Total versus normals		0.085	**0.0074**	0.2	**0.0006**	0.4194	0.1608
*P value SCD Mild versus Normals		0.632	**0.019**	**0.0471**	**0.006**	0.8143	**0.0064**
*P value SCD Severe versus Normals		**0.0172**	**0.0489**	0.8637	**0.03**	0.0682	1.000

*Chi-square test analysis comparing the Fc*γ*RIIA and Fc*γ*RIIIB gene frequencies in various groups.

**Table 5:** Comparison of expression of CD32 with different genotypes of Fc*γ*RIIA and CD16 with different genotypes of Fc*γ*RIIIB.

	SCD-mild (90)			SCD-severe (61)		
	H/H (18)	H/R (33)	R/R (39)	H/H (18)	H/R (23)	R/R (20)
	(Mean ± S.D.)	(Mean ± S.D.)	(Mean ± S.D.)	(Mean ± S.D.)	(Mean ± S.D.)	(Mean ± S.D.)
CD32 (MFI)	23.68 ± 13.24	39.21 ± 23.04	23.94 ± 15.85	15.49 ± 6.14	33.83 ± 19.96	27.32 ± 18.8

	NA1/NA2 (44)	NA2/NA2 (46)		NA1/NA2 (36)	NA2/NA2 (25)	

CD16 (MFI)	171.33 ± 85.08	162.22 ± 69.42		177.59 ± 88.61	206.48 ± 92.60	

Student's *t* test analysis comparing expression levels with different genotypes among mild versus severe SCD patients:

H/H: *P*-**0.023**; H/R: *P*-0.37; R/R: *P*-0.47.

NA1/NA2: *P*-0.75, NA2/NA2: *P*-**0.03**.

**Table 6:** Comparison of expression of neutrophil markers with vaso occlusive crises (VOC) in sickle cell disease patients.

CD Markers	Absence of VOC (51)	With episodes of VOC (97)	*P value
CD16 (MFI)	171.29 ± 71.05	177.52 ± 66.08	0.59
CD32 (MFI)	26.92 ± 19.48	28.05 ± 17.56	0.72
CD62L (MFI)	107.67 ± 29.98	92.31 ± 39.99	**0.019**

*Student's *t* test analysis comparing expression levels in patients with absence of vaso occlusive crises and with episodes of vaso occlusive crises.
